# Ecological Role of the Common Pheasant (*Phasianus colchicus*) in Maintaining Tick Populations in Croatia

**DOI:** 10.3390/ani16172712

**Published:** 2026-09-01

**Authors:** Krunoslav Pintur, Nera Fabijanić, Tomislav Dumić, Ema Gagović, Vedran Slijepčević, Daria Jurković Žilić, Relja Beck

**Affiliations:** 1 Department of Wildlife Management and Nature Conservation, Karlovac University of Applied Sciences, Trg J.J. Strossmayera 9, 47000 Karlovac, Croatia; tomislav.dumic@vuka.hr (T.D.); vedran.slijepcevic@vuka.hr (V.S.); 2 Croatian Hunting Association, Vladimira Nazora 63, 10000 Zagreb, Croatia; nera_az@yahoo.com; 3 Croatian Veterinary Institute, Savska cesta 143, PP 883, 10000 Zagreb, Croatia; gagovic@veinst.hr (E.G.); jurkovic@veinst.hr (D.J.Ž.)

**Keywords:** haplotype, *Haemaphysalis punctata*, *Ixodes acuminatus*, *Ixodes festai*, *Ixodes frontalis*, *Ixodes ricinus*, *Ixodes ventalloi*, molecular identification

## Abstract

Ticks are obligate hematophagous ectoparasites of public health importance, acting as vectors of numerous pathogens affecting wildlife, domestic animals and humans. Although birds play an important role in tick ecology and dispersal, information on tick infestations of pheasants in Europe remains limited. This study investigated the prevalence, species composition, developmental stage structure and haplotype diversity of ticks infesting pheasants in Croatia using ecological, morphological and molecular approaches. During the hunting seasons, 255 pheasants from continental and Mediterranean regions of Croatia were examined. Ticks were collected from harvested pheasants and road-killed individuals, morphologically identified, and representative specimens were molecularly confirmed. Overall, 213 pheasants were infested, resulting in a prevalence of 83.5%, and a total of 1103 ticks were collected. Six tick species were identified, with *Ixodes ricinus* dominating the tick community (80.1%). Molecular analyses confirmed the occurrence of several closely related Mediterranean *Ixodes* species, including *Ixodes festai* and *Ixodes ventalloi*. Nymphs predominated (78.2%), highlighting the importance of pheasants as hosts for immature tick stages. Significant geographical differences were observed. Continental localities were dominated by *I. ricinus*, whereas coastal and island regions showed significantly higher tick species diversity. Mean tick abundance per pheasant was significantly higher in Mediterranean regions. Male pheasants exhibited higher infestation prevalence and intensity than females. The marked regional differences highlight the influence of Croatia’s biogeographical heterogeneity on tick community composition. These findings demonstrate that pheasants serve as important hosts for diverse tick species and likely contribute to the maintenance of local tick populations, thereby providing a valuable baseline for future surveillance of tick-borne pathogens in Croatia.

## Simple Summary

Ticks are blood-feeding parasites that can transmit diseases to wildlife, domestic animals, and humans. Although birds play an important role in the life cycle and dispersal of ticks, little is known about tick infestations in common pheasants in Croatia. This study investigated the tick species infesting pheasants and evaluated their role as hosts for ticks. A total of 255 pheasants from different regions of Croatia were examined, and more than 80% were infested. More than 1100 ticks belonging to six species were collected, with *Ixodes ricinus* being the dominant species. Immature tick stages were especially abundant, indicating that pheasants are suitable hosts for tick development. Clear differences were observed between continental and Mediterranean Croatia, with Mediterranean localities harbouring a more diverse tick community and higher tick abundance. Male pheasants also carried significantly more ticks than females. These findings show that pheasants play an important role in maintaining local tick populations and provide new insights into tick ecology in Croatia. They also provide a valuable basis for future studies on tick-borne pathogens and their potential impact on wildlife, domestic animals, and human health.

## 1. Introduction

The common pheasant (*Phasianus colchicus*) is one of the most widespread and economically important gamebird species in Europe. Originally native to Asia, the species has been successfully introduced throughout much of Europe and today is a characteristic component of agricultural landscapes, lowland grasslands, forest edges and hunting grounds [1]. In Croatia, pheasants are widely distributed throughout lowland continental regions and constitute one of the most important small game species, playing a significant role in hunting, wildlife management and rural traditions. Due to their predominantly ground-dwelling behaviour, frequent use of field margins, grasslands, woodland edges and agricultural habitats, pheasants are continuously exposed to questing ticks and may represent important hosts for several tick species. Unlike migratory birds, pheasants are largely sedentary and typically occupy relatively small home ranges throughout the year. Consequently, their role in tick ecology is likely associated primarily with the maintenance of local host–tick cycles rather than long-distance dispersal of ticks and tick-borne pathogens. Similar patterns have been described for other resident galliform birds inhabiting European landscapes [2]. Birds are widely recognized as important hosts for immature stages of hard ticks and can contribute to both local maintenance and dispersal of tick populations and associated pathogens [2,3]. Numerous studies have demonstrated that birds may transport attached ticks between habitats and geographical regions, thereby influencing tick population dynamics and pathogen circulation [3,4]. Consequently, birds are increasingly recognized as important components of host–tick–pathogen systems throughout Europe [2]. Compared with migratory passerines, relatively little attention has been paid to galliform birds despite their potential importance as hosts for immature tick stages. Experimental and field studies conducted in the United Kingdom have shown that pheasants may harbour substantial tick burdens, predominantly larvae and nymphs of *Ixodes ricinus*, and that tick infestations can negatively affect territorial behaviour, breeding success and survival [5,6]. Furthermore, Kurtenbach et al. [7] demonstrated that pheasants are competent reservoir hosts for *Borrelia burgdorferi* sensu lato, indicating that this species may contribute not only to the maintenance of tick populations but also to pathogen circulation within natural ecosystems. Recent studies have further emphasized the importance of pheasants in vector-borne disease ecology. Michels et al. [8] demonstrated that the release of non-native gamebirds may amplify zoonotic disease risk by increasing host density and enhancing pathogen circulation within local tick populations. Such findings suggest that pheasant management practices may influence local tick–pathogen dynamics and should therefore be considered within a broader One Health framework [9,10]. In Croatia, *Ixodes ricinus* is the predominant tick species in continental lowland habitats and is widely distributed across forests, agricultural landscapes and habitat mosaics [11]. Previous studies have documented pronounced seasonal dynamics, habitat-related variation in tick abundance and widespread circulation of *B. burgdorferi* sensu lato and tick-borne encephalitis virus [12,13,14]. However, despite the ecological and epidemiological importance of pheasants, information regarding tick infestations in this species remains scarce in southeastern Europe. In Croatia, available data are currently limited to preliminary observations from lowland hunting areas [15], while comprehensive studies investigating infestation prevalence, tick species composition, developmental-stage structure and genetic diversity of pheasant-associated ticks are lacking. Given this lack of information, the aim of the present study was to investigate tick infestation in common pheasants in Croatia and to evaluate their potential role in the ecology of ticks inhabiting continental and Mediterranean environments. Specifically, the study assessed infestation prevalence, species composition, developmental-stage structure and haplotype diversity of ticks collected from pheasants across different geographical regions of Croatia, thereby improving our understanding of host–tick interactions and providing baseline data for future surveillance of tick-borne pathogens in Croatia.

## 2. Materials and Methods

### 2.1. Study Area

The common pheasant specimens included in this study were collected from localities situated within two major geographical regions of Croatia: continental Croatia and the Adriatic coastal and island region. Of the 255 examined birds, 227 originated from continental localities and 28 from coastal and island sites. Continental sampling sites were distributed across the Pannonian biogeographical region and included lowland agricultural landscapes, floodplain areas, mixed deciduous forests, cultivated fields and fragmented grasslands. In contrast, coastal and island localities were characterized by Mediterranean and sub-Mediterranean environmental conditions, including karst terrain, rocky grasslands, shrub-dominated vegetation, maquis communities and fragmented agricultural habitats. The island localities additionally represent geographically isolated ecosystems with distinct ecological conditions that may influence host–tick associations. The investigated localities encompassed two climatically distinct regions of Croatia. Continental Croatia is characterized by a temperate continental climate and lies within the zone of mid-latitude atmospheric circulation throughout the year. As a result, weather conditions are highly variable and frequently influenced by changing synoptic situations [16]. The Adriatic coastal and island region is influenced by mid-latitude circulation for much of the year; however, during summer it comes under the stronger influence of the Azores High, resulting in more stable weather conditions and reduced penetration of colder air masses. The Adriatic Sea acts as a major climatic regulator, moderating temperature extremes and contributing to the development of Mediterranean climatic conditions [16]. According to the Köppen climate classification, most continental localities belong to the humid temperate climate types Cfa and Cfb, whereas the coastal and island region is classified as Mediterranean climate with hot summers (Csa) [17].

### 2.2. Tick Sampling and Identification

Ticks were collected from harvested pheasants and road-killed individuals during 2024–2025. Specimens were morphologically identified to species, developmental stage, and sex using standard taxonomic keys [18,19,20]. Representative specimens were documented using a SteREO Discovery.V20 microscope (Carl Zeiss AG, Oberkochen, Germany) equipped with AxioVision and ZEN2 Pro software (blue edition, version 3.5.093.00010). After identification, each tick was washed in 70% ethanol, rinsed with sterile distilled water, dried on sterile filter paper to remove external contaminants (such as soil particles, host-derived material, feathers, and blood residues), and stored individually in sterile 2 mL tubes containing 96% ethanol until molecular analysis.

Species identification was based on the combined evaluation of morphological characteristics and mitochondrial 16S rRNA sequence similarity. A total of 244 representative specimens were selected for molecular confirmation. Specimens were chosen to ensure proportional representation of all morphologically identified species, developmental stages, and geographical regions, with particular emphasis on morphologically challenging taxa to validate species identification.

Genomic DNA was extracted using the MagMAX™ CORE Nucleic Acid Purification Kit (Thermo Fisher Scientific, Waltham, MA, USA) on the KingFisher™ Flex Purification System (Thermo Fisher Scientific, Waltham, MA, USA), according to the manufacturer’s instructions, with a final elution volume of 100 μL. Species identity was confirmed by amplification and sequencing of a 460 bp fragment of the mitochondrial 16S rRNA gene using the forward primer 16SF (5′-CTGCTCAATGTTTTTTAAATTGCTGTGG-3′) and reverse primer 16SR (5′-CCGGTCTGAACTCAGATCAAGT-3′) [21]. PCR reactions were performed in a final volume of 20 µL containing 10 µL GoTaq^®^ G2 Master Mix (Promega, Madison, WI, USA), 7.2 µL DNase/RNase-free distilled water (Promega, Madison, WI, USA), 0.4 µL of each primer (10 pmol/µL), and 2 µL DNA template. Positive and negative extraction controls were included in all amplification rounds to monitor contamination. Amplification products were evaluated by capillary electrophoresis using a QIAxcel system (Qiagen, Hilden, Germany) with the QIAxcel DNA Fast Analysis kit (Qiagen, Hilden, Germany), DNA QX Alignment Marker (15 bp–3 kb; Qiagen, Hilden, Germany), and QX DNA Size Markers (50 bp–1.5 kb and 100 bp–2.5 kb; Qiagen, Hilden, Germany). PCR products were purified using ExoSAP-IT™ PCR Product Clean-up Reagent (Applied Biosystems™, Waltham, MA, USA) according to the manufacturer’s instructions and sequenced bidirectionally at Macrogen Europe using the corresponding primer sets. Raw sequence chromatograms were assembled and edited using SeqMan Pro 18 and SeqBuilder Pro 18 (DNASTAR, Madison, WI, USA). Consensus sequences were aligned and analysed using SeqMan and SeqBuilder Pro version 18 within the Lasergene 18.6 software suite (DNASTAR, Madison, WI, USA) and compared with reference sequences available in GenBank using BLASTn against the GenBank database (NCBI).

For haplotype analysis, 16S rRNA sequences assigned to the same species were aligned and trimmed to a common overlapping fragment. Sequences showing identical nucleotide composition across the analysed fragment were assigned to the same haplotype, whereas sequences differing by one or more nucleotide substitutions were considered different haplotypes. Haplotype labels (H1, H2, etc.) were assigned separately for each tick species. No phylogenetic analyses were performed; therefore, haplotypes represent sequence variants identified within the analysed 16S rRNA fragment rather than inferred evolutionary lineages.

### 2.3. Statistical Analysis

Statistical analyses were performed using the open-source statistical software R version 4.5.1 (R Foundation for Statistical Computing, Vienna, Austria). Data were checked, compiled, and summarized in Microsoft Excel, and all statistical analyses were subsequently performed in R.

Parasitological descriptors followed the definitions of Bush et al. [22]. Tick prevalence was defined as the percentage of examined pheasants infested with one or more ticks, whereas mean intensity was calculated as the average number of ticks per infested pheasant. Mean abundance was defined as the average number of ticks per examined pheasant. Tick prevalence is presented as percentages with corresponding Wilson 95% confidence intervals (95% CI).

Because parasite count data are typically aggregated and right-skewed, non-parametric methods were used for univariable comparisons, whereas generalized linear models (GLMs) with appropriate error distributions were applied for multivariable analyses [23].

Differences in infestation prevalence among sampling localities, geographical regions, and host sexes were evaluated using Pearson’s chi-square (χ^2^) test. Differences in infestation intensity and tick abundance among sampling localities, geographical regions, and host sexes were assessed using the Kruskal–Wallis test (H).

The distribution of tick species among sampling localities and geographical regions was compared using Pearson’s χ^2^ tests. Developmental-stage composition was analysed both overall and separately for each tick species, and associations between tick species and developmental stages were evaluated using Pearson’s χ^2^ tests.

Haplotype frequencies were calculated separately for each tick species. For each species, haplotype distribution among sampling localities and geographical regions was analysed to assess geographical clustering and population structuring. Differences in haplotype frequencies among localities and geographical regions were evaluated using Pearson’s χ^2^ tests.

To evaluate the simultaneous effects of host sex, geographical region, and sampling season on tick infestation, generalized linear models (GLMs) were fitted. Tick infestation prevalence was analysed using a binomial GLM with a logit link function, whereas tick abundance was analysed using a negative binomial GLM to account for overdispersion of count data. Model results are presented as odds ratios (OR) or incidence rate ratios (IRR) with corresponding 95% confidence intervals. All statistical tests were two-tailed, and statistical significance was accepted at *p* < 0.05.

## 3. Results

A total of 255 pheasants were examined for tick infestation, of which 213 were infested, resulting in an overall infestation prevalence of 83.5% (95% CI: 78.5–87.6%). A total of 1103 ticks were collected. The mean infestation intensity was 5.18 ticks per infested pheasant (median = 3; range = 1–30), whereas the mean abundance was 4.33 ticks per examined pheasant (median = 3; range = 0–30). Overall epidemiological descriptors are summarized in Table 1. Mixed-species infestations were recorded in 25 pheasants (9.8%). Six tick species were identified. *Ixodes ricinus* accounted for 80.1% of all collected ticks, followed by *Ixodes frontalis* (12.0%), *Ixodes festai* (5.5%), *Ixodes ventalloi* (1.9%), *Haemaphysalis punctata* (0.4%), and *Ixodes acuminatus* (0.1%). The frequencies of collected tick species differed significantly (χ^2^ = 3982.28, df = 6, *p* < 0.001). The developmental-stage composition differed significantly among tick species (χ^2^ = 604.33, df = 10, *p* < 0.001) and is presented in Table 2. Nymphs predominated in *I. ricinus* and *I. frontalis*, whereas adults represented a relatively larger proportion of *I. festai* and *I. ventalloi*. In contrast, *H. punctata* was represented almost exclusively by predominantly by nymphs. These findings indicate marked interspecific differences in developmental-stage structure (Table 2).

Infestation prevalence differed significantly among sampling localities (χ^2^ = 55.50, df = 17, *p* < 0.001). Tick abundance also varied significantly among localities (Kruskal–Wallis H = 78.18, *p* < 0.001), indicating substantial spatial variation in host exposure to ticks. Comparisons between geographical regions and host sexes are summarized in Table 3. When grouped into geographical regions, infestation prevalence was numerically higher in coastal and island localities (95.2%) than in continental Croatia (82.5%), although the difference was not statistically significant (χ^2^ = 2.28, df = 1, *p* = 0.131). However, tick abundance was significantly higher in coastal and island regions (mean abundance 5.90 ticks per examined bird, 95% CI: 3.86–7.52) than in continental regions (mean abundance 4.21 ticks per examined bird, 95% CI: 3.59–4.88) (Kruskal–Wallis H = 4.31, *p* = 0.038). Sex-related differences were also evident. Male pheasants had a significantly higher prevalence (193/225; 85.8%; 95% CI: 80.6–89.7%) than females (20/30; 66.7%; 95% CI: 48.8–80.8%) (χ^2^ = 7.03, df = 1, *p* = 0.008; OR = 3.02, 95% CI: 1.29–7.03). Mean intensity was higher in males than females (5.46 vs. 2.50 ticks per infested bird; Kruskal–Wallis H = 8.03, *p* = 0.005). Mean abundance showed the same pattern (4.68 vs. 1.67 ticks per examined bird; Kruskal–Wallis H = 13.46, *p* < 0.001).

**Figure 1:**
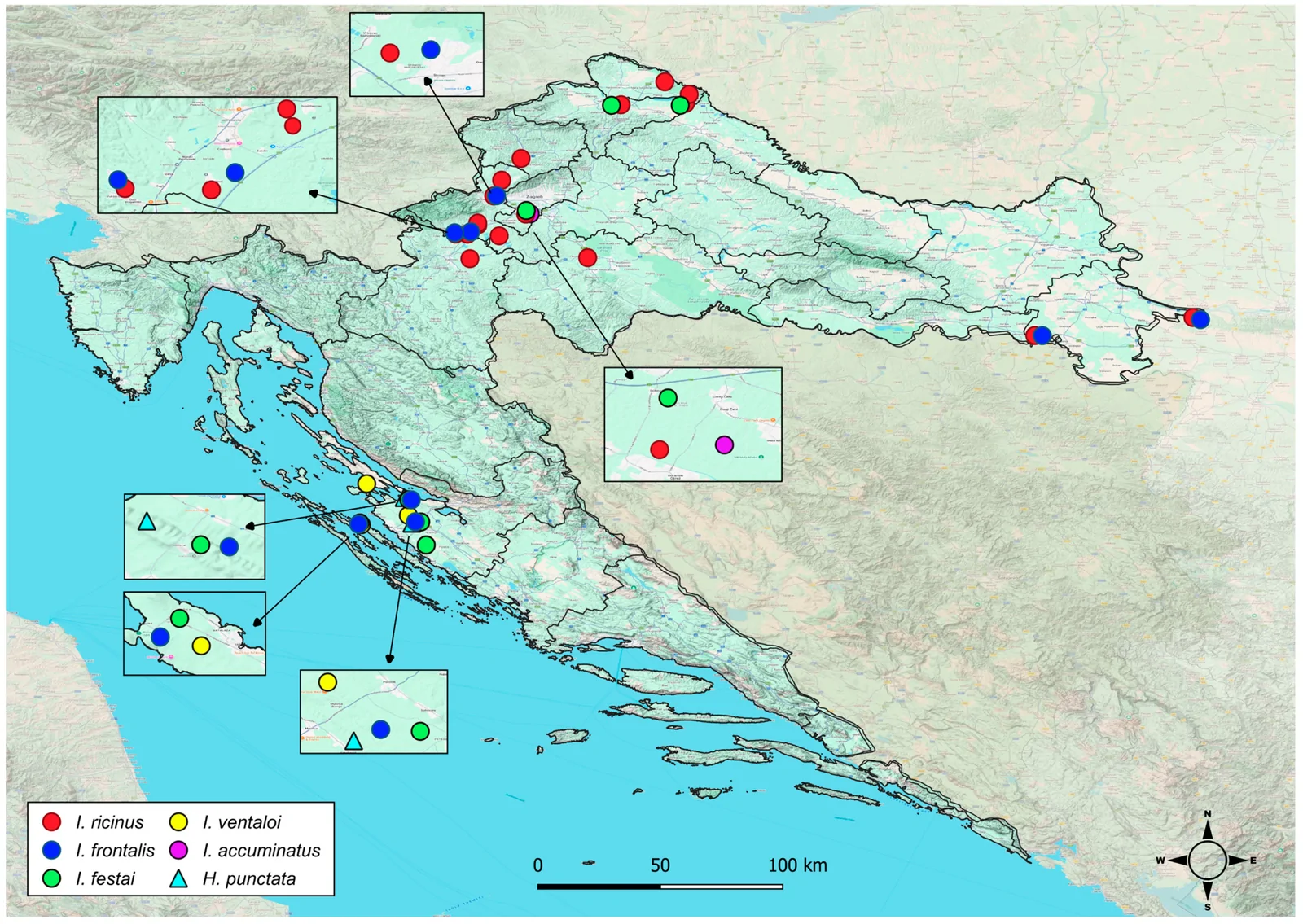
Geographic distribution of tick species collected from common pheasants across Croatia during 2024–2025. Each coloured symbol indicates the presence of a tick species at a sampling locality rather than the number of collected specimens. Insets show enlarged views of areas where sampling localities are closely spaced.

Species composition differed significantly among localities (χ^2^ = 1337.06, df = 102, *p* < 0.001) and between geographical regions (χ^2^ = 789.82, df = 5, *p* < 0.001). Continental localities were dominated by *I. ricinus*, whereas *I. ricinus* was not detected on pheasants from the coastal and island localities. Instead, these localities were characterized primarily by *I. festai* and *I. ventalloi*, reflecting a distinct tick species composition (Figure 1). This regional differentiation indicates the presence of distinct tick assemblages associated with different ecological and climatic zones (Figure 1).

To assess the simultaneous effects of sex, geographical region, and sampling season on tick infestation, generalized linear models were fitted. In the binomial GLM, sex and sampling season were significantly associated with infestation prevalence, whereas geographical region was not. After adjustment for region and season, female pheasants had significantly lower odds of infestation than males (OR = 0.30, 95% CI: 0.11–0.83, *p* = 0.021). Pheasants sampled during winter also had significantly lower odds of infestation than those sampled during autumn (OR = 0.08, 95% CI: 0.03–0.17, *p* < 0.001). Geographical region was not significantly associated with infestation prevalence (coastal/island vs. continental region: OR = 1.20, 95% CI: 0.14–9.87, *p* = 0.868). The effect of spring could not be estimated reliably because only two pheasants were sampled during this season and both were infested.

A negative binomial GLM was used to evaluate the simultaneous effects of sex, geographical region, and sampling season on tick abundance. Female pheasants harboured significantly fewer ticks than males (IRR = 0.37, 95% CI: 0.24–0.57, *p* < 0.001). Tick abundance was also significantly lower during winter than during autumn (IRR = 0.28, 95% CI: 0.20–0.39, *p* < 0.001). Geographical region was not a significant predictor of tick abundance (coastal/island vs. continental region: IRR = 1.02, 95% CI: 0.67–1.56, *p* = 0.921). Although tick abundance appeared higher during spring than during autumn, this difference was not statistically significant (IRR = 2.91, 95% CI: 0.87–9.69, *p* = 0.082) and should be interpreted cautiously because only two pheasants were sampled in spring.

The highest number of haplotypes was observed in *I. ricinus*, in which eight distinct haplotypes were detected (Table A1). Haplotype H7 was the most prevalent lineage (48/190; 25.3%), followed by H8 (40/190; 21.1%), H2 (36/190; 18.9%), H1 (29/190; 15.3%), H4 (20/190; 10.5%), H6 (13/190; 6.8%), H10 (4/190; 2.1%) and H11 (2/190; 1.1%) (Table 1). Several haplotypes co-occurred within the same localities, indicating the coexistence of multiple genetic lineages within local populations. Three haplotypes were identified in *I. frontalis*, of which H1 predominated strongly (20/28; 71.4%). Haplotype H2 was detected in 5 specimens (17.9%), whereas H3 occurred only sporadically (3 specimens; 10.7%). Four haplotypes were recorded in *I. festai* (Table 1). H1 (6/18; 33.3%), H3 (5/18; 27.8%) and H5 (5/18; 27.8%) were the dominant lineages. Haplotype H6 (2 specimens; 11.1%) was the least prevalent. The presence of multiple haplotypes within geographically restricted Mediterranean localities suggests local diversification and long-term persistence of populations. A single haplotype was identified in *I. ventalloi* (*n* = 5), and *I. acuminatus* (*n* = 1). These findings indicate substantially lower genetic variability compared with *I. ricinus* and *I. festai*. The geographical distribution of haplotypes revealed pronounced population structuring. *I. ricinus* was restricted to continental localities, whereas *I. ventalloi* occurred exclusively in the coastal region. Most *I. festai* haplotypes were likewise associated with Mediterranean localities. Consequently, haplotype frequencies differed significantly among localities (χ^2^ = 3017.29, df = 289, *p* < 0.001) and between geographical regions (χ^2^ = 802.63, df = 17, *p* < 0.001). Seasonal analyses showed no significant differences in overall tick species composition among seasons (χ^2^ = 8.26, df = 6, *p* = 0.220). However, developmental-stage composition varied significantly (χ^2^ = 7.37, df = 2, *p* = 0.025), indicating seasonal shifts in the relative abundance of larvae, nymphs and adults. Nymphs predominated throughout the year, whereas larvae were relatively more frequent during warmer months.

## 4. Discussion

To our knowledge, this study represents the first comprehensive investigation of tick infestation in common pheasants in Croatia and one of the few European studies combining ecological, morphological, and molecular approaches to characterize the composition of tick species associated with this host species. The high overall infestation prevalence of 83.9%, together with the collection of more than 1100 ticks belonging to six species, demonstrates that pheasants are frequently exposed to ticks and support a considerably more diverse tick assemblage than might be expected from the limited data currently available for this gamebird in Europe [2]. Rather than representing occasional hosts, pheasants appear to be regular hosts for several tick species and may contribute to the maintenance of local tick populations.

Although the common pheasant is native to Asia, published information on tick infestations in wild pheasant populations within its native range remains surprisingly limited. Available studies from East Asia have only demonstrated that pheasants can serve as hosts for ixodid ticks, whereas comprehensive ecological investigations comparable to the present study are still lacking [24,25].

As expected, *I. ricinus* dominated the tick community, accounting for 80% of all collected specimens. This species is the most widespread and epidemiologically important tick in Europe and is characterized by a broad host range and pronounced ecological plasticity [2,19,26]. Its predominance on pheasants is therefore consistent with its widespread occurrence in continental Croatian habitats [11,27,28,29,30]. Nevertheless, the identification of five additional tick species is particularly noteworthy. In addition to *I. ricinus*, pheasants were infested with *H. punctata*, *I. acuminatus*, *I. festai*, *I. frontalis* and *I. ventalloi*.

The occurrence of *I. frontalis* is consistent with its well-established association with birds and its widespread occurrence on avian hosts in Europe [2]. In contrast, the molecular confirmation of *I. festai* and *I. ventalloi* deserves particular attention. The genus *Ixodes* is the most species-rich tick genus, with 291 recognised species, including 47 described between 2000 and 2025 [31]. The continuing discovery of new species reflects the taxonomic complexity of the genus, where closely related species often show only subtle morphological differences and molecular reference data remain limited. Recent taxonomic reassessments have further demonstrated that the number of *Ixodes* species occurring in the Mediterranean region has probably been underestimated and that morphologically similar or cryptic taxa may occur within this group [20]. In this context, molecular confirmation of *I. festai* and *I. ventalloi* from pheasants is particularly relevant and provides important new information on their host associations and geographical distribution.

The relatively high number of *I. festai* collected in the present study is particularly noteworthy. This species has traditionally been regarded primarily as a tick of birds, especially passeriform species, but its distribution, host spectrum, and morphological distinction from closely related *Ixodes* species remain incompletely understood [18,20]. A total of 61 specimens were identified, making it the third most abundant tick species found on pheasants and, to our knowledge, representing the first report of *I. festai* from common pheasants [2]. Its repeated occurrence, together with molecular confirmation of representative specimens, provides strong evidence that common pheasants are suitable hosts for this tick species in Mediterranean Croatia. Furthermore, the detection of four *I. festai* haplotypes among the sequenced specimens indicates the presence of multiple genetic variants within the studied population.

The detection of *I. ventalloi* further expands the number of tick species recorded on common pheasants. Although *I. ventalloi* has traditionally been regarded as a “rabbit tick” primarily associated with lagomorphs, its known host range also includes carnivores, rodents, and birds [19,31,32]. Increasing evidence indicates that avian hosts may play a more important role in its ecology than previously recognised, with different developmental stages reported from several bird orders, including Galliformes [33]. Common pheasants have previously been reported among the avian hosts of *I. ventalloi* [34]; however, such records remain scarce, and the ecological significance of this host association is still poorly understood.

Molecular confirmation of *I. ventalloi* was particularly relevant because this species may be difficult to distinguish morphologically from closely related Ixodes species, especially *I. festai* [30]. Recent molecular studies have demonstrated considerable genetic heterogeneity within *I. ventalloi* and identified distinct genogroups accompanied by subtle morphological differences in characters such as the auriculae and coxa I [35].

These observations further support the use of molecular methods alongside morphological identification, particularly when investigating closely related Mediterranean *Ixodes* species. The detection of *I. ricinus*, *I. festai*, and *I. ventalloi* on a single avian host species illustrates the complexity of Mediterranean Ixodes assemblages and highlights the value of combining detailed morphological examination with molecular identification.

An important ecological aspect of the present findings is the largely sedentary behaviour of common pheasants. Previous reports of *I. ventalloi* from migratory birds have raised the possibility that birds may transport this tick beyond its established range [35]. The recent detection of *I. ventalloi* on migratory Dunnocks in Slovakia was similarly interpreted as a possible consequence of avian migration [34]. In contrast, common pheasants generally occupy relatively restricted home ranges. Therefore, the repeated detection of *I. ventalloi* and *I. festai* on pheasants from Mediterranean and island localities in Croatia is unlikely to result from long-distance host-mediated dispersal. Instead, these findings more likely reflect established local tick populations and repeated host–tick interactions within these ecosystems [36]. Pheasants may therefore be particularly informative hosts for characterising local tick communities, as ticks collected from them are more likely to reflect exposure within the surrounding habitat.

The developmental-stage structure of the collected tick population provides additional support for the ecological importance of pheasants as hosts. Larvae and nymphs together accounted for more than 95% of all collected specimens, with nymphs alone representing 78.2%. This predominance of immature stages suggests that pheasants regularly serve as hosts for developmental stages that are essential for completion of the tick life cycle. Therefore, pheasants should not be regarded merely as incidental hosts carrying occasional adult ticks. Instead, they appear to support the immature stages of several tick species and may consequently contribute to the maintenance of local tick populations. This is particularly evident for *I. ricinus* and *I. frontalis*, in which nymphs predominated, whereas the higher proportion of adult *I. festai* and *I. ventalloi* indicates differences in host use among species.

One of the clearest findings of the present study was the pronounced geographical differentiation of pheasant-associated tick communities. Continental localities were dominated by *I. ricinus*, whereas *I. ricinus* was not detected on pheasants from the coastal and island localities. Instead, these localities were characterized primarily by *I. festai* and *I. ventalloi*, reflecting a distinct tick species composition. The absence of *I. ricinus* on pheasants from the Mediterranean localities is noteworthy, as this species is known to occur in parts of the Croatian Adriatic region [11,12]. Recent distribution maps indicate that *I. ricinus* is widespread throughout continental Croatia but has a more restricted and patchy distribution along the Adriatic coast, where its occurrence is largely confined to humid and forested habitats [11]. In contrast, the Mediterranean localities included in the present study are characterized predominantly by open karst landscapes, shrub vegetation, and dry Mediterranean climatic conditions, which are generally less favourable for *I. ricinus*. Under these environmental conditions, pheasants appear to be exposed primarily to Mediterranean *Ixodes* species such as *I. festai* and *I. ventalloi*. This marked geographical differentiation indicates the presence of distinct tick species associated with contrasting ecological and climatic zones in Croatia.

Tick abundance was also significantly higher in Mediterranean localities. Croatia occupies a biogeographically complex position at the transition between continental Central Europe and the Mediterranean, with pronounced differences in climate, vegetation, and habitat structure over relatively short geographical distances [16,17]. The observed geographical differences therefore suggest that this environmental heterogeneity may contribute substantially to shaping the species composition of local tick communities.

Haplotype analysis further supported the geographical differences observed in tick species distribution. Four *I. festai* haplotypes were identified among 18 sequences, whereas only a single haplotype was detected in *I. ventalloi* and *I. acuminatus*. However, the limited number of sequences available for these species precludes broader conclusions regarding the number, distribution, and geographical structuring of their haplotypes. The predominantly continental distribution of *I. ricinus* haplotypes and the Mediterranean occurrence of *I. festai* and *I. ventalloi* were consistent with the regional differences in tick species composition. Further studies using larger sample sizes and additional genetic markers are needed to better understand the population structure of these species.

The interpretation of haplotype H11 requires particular caution [31,37,38]. Although the H11 sequences were identical to a GenBank reference sequence deposited as *I. inopinatus*, they were assigned to *I. ricinus* in the present study. This interpretation was based on morphological identification and recent taxonomic evidence indicating that European sequences previously assigned to *I. inopinatus* are likely to represent *I. ricinus* [31,38]. Accordingly, these sequences were included within *I. ricinus* as haplotype H11 (Table 4).

**Figure 2:**
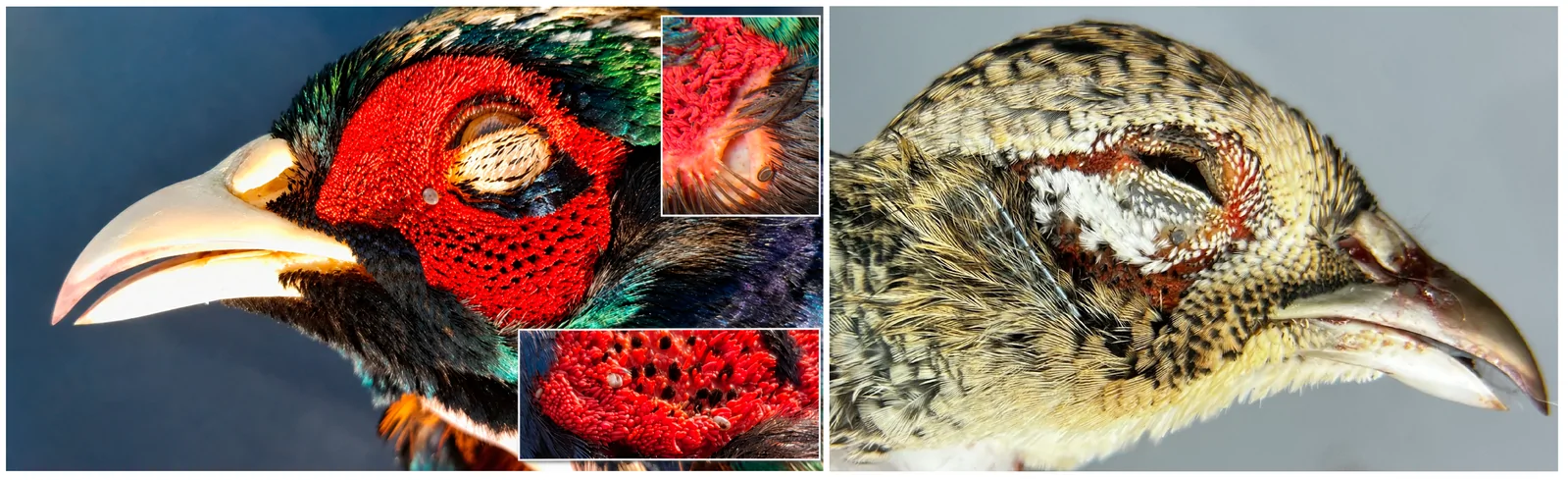
Representative attachment sites of ticks on common pheasants. Ticks were observed exclusively on the head region, particularly around the beak, eyes and ears.

Male pheasants exhibited significantly higher infestation prevalence and harboured more ticks than females. This difference may be related to a combination of anatomical, physiological, and behavioural characteristics. Most ticks were attached to featherless areas of the head, particularly the wattle beneath the beak and the regions surrounding the eyes and ears (Figure 2). The highly vascularized wattle provides an accessible feeding site, and its substantially larger size in males may increase the availability of suitable attachment areas. In addition, male pheasants exhibit pronounced territorial behaviour and greater movement, particularly during the breeding season, potentially increasing their exposure to questing ticks. Physiological differences may also contribute to this pattern. Experimental studies have demonstrated that testosterone can suppress both innate and acquired resistance to tick infestation, thereby increasing tick attachment success and feeding performance [39,40,41]. Although testosterone levels were not measured in the present study, hormone-mediated differences in immune function may partly explain the higher tick burden observed in males. Further studies are needed to determine the relative contributions of anatomical, behavioural, and physiological factors to this sex-related difference.

Previous studies have primarily emphasized the importance of pheasants as hosts for *I. ricinus*, particularly in the United Kingdom, where substantial burdens of immature ticks have been associated with changes in territorial behaviour, breeding success, and survival [5,6]. Experimental studies have additionally demonstrated the reservoir competence of pheasants for *Borrelia burgdorferi* sensu lato, indicating that their ecological role may extend beyond supporting tick development to involvement in pathogen circulation [7]. More recently, increased densities of released gamebirds have been associated with the amplification of local tick and zoonotic pathogen cycles [8]. However, most available studies have focused predominantly on *I. ricinus*, and comparatively little is known about the broader range of tick species capable of exploiting pheasants as hosts. The present findings therefore substantially expand the number of tick species known to parasitize common pheasants and demonstrate that the ecological relationship between pheasants and ticks is considerably more complex than previously recognized.

From a broader ecological perspective, the present findings demonstrate that common pheasants can support a diverse tick community and may contribute differently to tick population dynamics in continental and Mediterranean environments. Their ground-dwelling behaviour increases contact with questing ticks, while their largely sedentary lifestyle makes them particularly relevant to local host–tick interactions. The high infestation prevalence, predominance of immature developmental stages, and repeated occurrence of several *Ixodes* species indicate that pheasants are more than incidental tick hosts. At the same time, the pronounced regional differences in tick species composition show that the role of the same host species may differ considerably between contrasting climatic and ecological environments.

Several limitations should be considered when interpreting these results. Sampling intensity differed among localities and between geographical regions, with substantially fewer pheasants examined from coastal and island sites than from continental Croatia. Consequently, direct comparisons of the number of tick species and the number and distribution of haplotypes between regions should be interpreted with caution. In addition, the study focused primarily on tick species composition and population structure, while screening for tick-borne pathogens was beyond its scope. Nevertheless, the large number of examined pheasants and collected ticks, together with extensive molecular confirmation of morphologically identified specimens, provides a robust baseline for future studies of pheasant-associated tick communities and their potential epidemiological significance.

## 5. Conclusions

In conclusion, this study provides the first comprehensive investigation of tick species associated with common pheasants in Croatia and demonstrates that pheasants serve as important hosts for multiple tick species. Significant differences in species composition, infestation intensity, and haplotype distribution were detected between continental and Mediterranean regions, highlighting the influence of Croatia’s environmental heterogeneity on tick ecology. Furthermore, the pronounced geographical structuring of tick haplotypes suggests that continental and Mediterranean tick populations represent distinct ecological units. These findings contribute substantially to our understanding of tick species composition, host associations, population structure, and tick ecology in southeastern Europe.

## Figures and Tables

**Table 1:** Epidemiological descriptors of tick infestation on common pheasants according to geographical region and host sex.

Variable	Examined (*n*)	Infested (*n*)	Prevalence % (95% CI)	Mean Intensity (95% CI)	Mean Abundance (95% CI)
Overall	255	213	83.5 (78.5–87.6)	5.18 (4.54–5.86)	4.33 (3.74–4.95)
Continental Croatia	234	193	82.5 (77.1–86.8)	5.10 (4.42–5.85)	4.21 (3.59–4.87)
Coastal and island Croatia	21	20	95.2 (77.3–99.2)	5.90 (4.15–7.85)	5.62 (3.90–7.52)
Male pheasants	225	193	85.8 (80.6–89.7)	5.46 (4.75–6.22)	4.68 (4.03–5.38)
Female pheasants	30	20	66.7 (48.8–80.8)	2.50 (1.80–3.35)	1.67 (1.07–2.37)

**Table 2:** Developmental-stage composition of tick species collected from common pheasants in Croatia.

Tick Species	Larvae *n* (%)	Nymphs *n* (%)	Adults *n* (%)	Total *n* (%)
*Ixodes ricinus*	78 (8.8)	802 (90.7)	4 (0.5)	884 (80.1)
*Ixodes frontalis*	90 (68.2)	30 (22.7)	12 (9.1)	132 (12.0)
*Ixodes festai*	25 (41.0)	17 (27.9)	19 (31.1)	61 (5.5)
*Ixodes ventalloi*	1 (4.8)	10 (47.6)	10 (47.6)	21 (1.9)
*Haemaphysalis punctata*	0 (0.0)	3 (75.0)	1 (25.0)	4 (0.4)
*Ixodes acuminatus*	0 (0.0)	0 (0.0)	1 (100.0)	1 (0.1)
Total	194 (17.6)	862 (78.2)	47 (4.3)	1103 (100.0)

**Table 3:** Summary of statistical analyses of tick infestation prevalence, abundance, intensity, species composition, and developmental-stage composition in relation to locality, geographical region, sex, and tick species.

Response Variable	Comparison	Test	Statistic	df	*p*
Infestation prevalence	Localities	Pearson χ^2^	55.50	17	<0.001
Infestation prevalence	Regions	Pearson χ^2^	2.28	1	0.131
Infestation prevalence	Sex	Pearson χ^2^	7.03	1	0.008
Tick abundance	Localities	Kruskal–Wallis (H)	78.18	17	<0.001
Tick abundance	Regions	Kruskal–Wallis (H)	4.30	1	0.038
Tick abundance	Sex	Kruskal–Wallis (H)	13.46	1	<0.001
Infestation intensity	Localities	Kruskal–Wallis (H)	76.71	17	<0.001
Infestation intensity	Regions	Kruskal–Wallis (H)	1.85	1	0.174
Infestation intensity	Sex	Kruskal–Wallis (H)	8.03	1	0.005
Tick species composition	Localities	Pearson χ^2^	1337.06	102	<0.001
Tick species composition	Regions	Pearson χ^2^	789.82	5	<0.001
Developmental-stage composition	Tick species	Pearson χ^2^	604.33	10	<0.001

**Table 4:** Molecular identification of tick haplotypes based on mitochondrial 16S rRNA sequences, sequence identity, and GenBank accession numbers.

Tick Species (Haplotype)	Per. Ident. (%)	Acc. Number	Our Acc. Number
*I. ricinus* (H1)	100	PZ600149	PZ652811
*I. ricinus* (H2)	100	PZ600150	PZ652812
*I. ricinus* (H4)	100	PV113054	PZ652813
*I. ricinus* (H6)	100	ON116372	PZ652814
*I. ricinus* (H7)	100	PZ600151	PZ652815
*I. ricinus* (H8)	100	PZ600152	PZ652816
*I. ricinus* (H10)	100	PZ600153	PZ652817
*I. ricinus* (H11) *	100	GU074596	PZ652809
*I. festai* (H1)	100	PZ599990	PZ652805
*I. festai* (H3)	100	PZ599992	PZ652806
*I. festai* (H5)	100	PZ599994	PZ652807
*I. festai* (H6)	100	PZ599995	PZ652808
*I. frontalis* (H1)	99.6	PQ772137	PZ649543
*I. frontalis* (H2)	100	KJ414454	PZ649544
*I. frontalis* (H3)	99.7	PQ772137	PZ649545
*I. acuminatus*	100	PZ597521	PZ652804
*I. ventalloi* (H2)	100	PZ600181	PZ652810

* *I. ricinus* (H11) showed 100% sequence identity to GenBank sequences previously annotated as *I. inopinatus*, which is currently synonymised with *I. ricinus* and not regarded a valid species [31,38].

## Data Availability

The nucleotide sequences generated in this study have been deposited in the GenBank database under the accession numbers provided in the manuscript (PZ652804–PZ652817; PZ649543–PZ649545). The datasets supporting the findings of this study are available from the corresponding author upon reasonable request.

## Appendix A

**Table A1:** Sampling localities, geographic coordinates, and tick species haplotypes identified in common pheasants (*Phasianus colchicus*) in Croatia.

Locality	GPS Coordinates	Tick Species (Haplotypes)
Jastrebarsko	45°40′44.5″ N 15°41′23.2″ E	*I. ricinus* (H1, H2, H4, H6, H7, H8, H10, H11); *I. frontalis* (H2, H3)
Sveta Nedelja	45°49′14.2″ N 15°46′29.5″ E	*I. ricinus* (H1, H2, H6, H7, H8); *I. frontalis* (H2, H3)
Pojatno	45°53′59.2″ N 15°48′44.9″ E	*I. ricinus* (H2, H7)
Zabok	46°00′40.8″ N 15°54′43.5″ E	*I. ricinus* (H8)
Bratina	45°36′50.4″ N 15°47′58.9″ E	*I. ricinus* (H7)
Novi Zagreb	45°43′29.7″ N 15°57′32.4″ E	*I. ricinus* (H1, H2, H4, H6, H7, H10); *I. accuminatus*; *I. festai* (H3)
Hodošan	46°24′19.6″ N 16°39′01.5″ E	*I. ricinus* (H2, H7, H8)
Dubrava	46°20′27.4″ N 16°46′34.2″ E	*I. ricinus* (H6, H8)
Mali Bukovec	46°17′23.6″ N 16°45′00.8″ E	*I. ricinus* (H6, H7), *I. festai* (H3)
Varaždin	46°16′41.6″ N 16°24′05.3″ E	*I. ricinus* (H1, H2, H8), *I. festai* (H5)
Rečica	45°29′47.3″ N 15°38′57.5″ E	*I. ricinus* (H1, H2, H4, H7, H8, H10)
Mala Gorica	45°30′06.3″ N 16°15′16.1″ E	*I. ricinus* (H2, H6, H7)
Volavje	45°37′31.6″ N 15°34′45.6″ E	*I. ricinus* (H1, H2, H4, H6, H7, H8), *I. frontalis* (H1)
Babina Greda	45°06′15.0″ N 18°34′03.3″ E	*I. ricinus* (H1), *I. frontalis* (H1)
Ilok	45°11′36.6″ N 19°22′46.4″ E	*I. ricinus* (H1, H2, H4, H6, H7, H8), *I. frontalis* (H1, H2)
Raštane	44°01′31.6″ N 15°25′29.6″ E	*I. festai* (H1, H3, H5)
Pag	44°20′24.4″ N 15°07′09.0″ E	*I. ventalloi* (H2)
Ražanac	44°15′29.5″ N 15°20′06.6″ E	*I. festai* (H6), *I. frontalis* (H1, H3), *H. punctata*
Ugljan	44°07′46.6″ N 15°05′30.7″ E	*I. festai* (H1, H3, H5), *I. ventalloi* (H2), *I. frontalis* (H1)
Zadar	44°08′44.4″ N 15°22′09.0″ E	*I. festai* (H3, H5, H6), *I. frontalis* (H1), *H. punctata*, *I. ventalloi* (H2)
